# Full-length genome sequences of the first H9N2 avian influenza viruses isolated in the Northeast of Algeria

**DOI:** 10.1186/s12985-020-01377-z

**Published:** 2020-07-17

**Authors:** Abdelheq Barberis, Amine Boudaoud, Angelina Gorrill, Josianne Loupias, Abdeljelil Ghram, Jihene Lachheb, Nadir Alloui, Mariette F. Ducatez

**Affiliations:** 1 Centre de Recherche en Biotechnologie, Nouvelle Ville Ali Mendjeli, El Khroub, Algeria; 2 LESPA, Département vétérinaire, ISVSA, Université de Batna, Batna, Algeria; 3 IHAP, Université de Toulouse, INRAE, ENVT, 23 Chemin des Capelles, 31076 Toulouse cedex, France; 4 Laboratoire d’Epidémiologie et de Microbiologie Vétérinaire, Institut Pasteur de Tunis, Université Tunis El Manar, Tunis, Tunisia

**Keywords:** Avian influenza H9N2, Algeria, Full-length genome sequencing, Phylogenetic analysis, Molecular characterization

## Abstract

**Background:**

H9N2 avian influenza viruses (AIV) has a worldwide geographic distribution and affects poultry of different types of production. H9N2 AIV was first reported in the Northeast of Algeria in April 2017, following an outbreak associated with high mortality, in broiler flocks. In the present study, we report full-length genome sequences of AIV H9N2, and the detailed phylogeny and molecular genetic analyses.

**Methods:**

Ten AIV H9N2 strains, collected in broiler flocks, were amplified in 9-day-old embryonated specific pathogen free (SPF) chicken eggs. Their full-length genomes were successfully sequenced and phylogenetic and molecular characterizations were conducted.

**Results:**

Phylogenetic analysis showed that the isolates were monophyletic, grouped within the G-1 lineage and were very close to Moroccan and Algerian strains identified in 2016 and 2017, respectively. The low pathogenicity of the strains was confirmed by the sequence motif (335RSSR/GLF341) at the hemagglutinin (HA) cleavage site. An exclusive substitution (T197A) that had not been previously reported for H9N2 viruses; but, conserved in some pandemic H1N1 viruses, was observed. When compared to the G1-like H9N2 prototype, the studied strains showed one less glycosylation site in HA, but 2–3 additional ones in the stalk of the neuraminidase (NA). The HA protein harbored the substitution 234 L, suggesting binding preference to human-like receptors. The NA protein harbored S372A and R403W substitutions, previously detected in H9N2 from Asia and the Middle East, and especially in H2N2 and H3N2 strains that caused human pandemics. Different molecular markers associated with virulence and mammalian infections have been detected in the viral internal proteins. The matrix M2 protein possessed the S31N substitution associated with drug resistance. The non-structural 1 (NS1) protein showed the “GSEV” PDZ ligand (PL) C-terminal motif and no 80–84 deletion.

**Conclusion:**

Characterized Algerian AIV isolates showed mutations that suggest increased zoonotic potential. Additional studies in animal models are required to investigate the pathogenicity of these H9N2 AIV strains. Monitoring their evolution in both migratory and domestic birds is crucial to prevent transmission to humans. Implementation of adequate biosecurity measures that limit the introduction and the propagation of AIV H9N2 in Algerian poultry farm is crucial.

## Background

Avian influenza (AI) is an infectious viral disease that mainly affects the respiratory or digestive system. The avian influenza viruses (AIV) affect different species of wild and domestic birds [1]. They belong to the *Orthomyxoviridae* family, having a single stranded RNA genome composed of eight segments that code for more than 11 viral proteins. The Hemagglutinin (HA) and the neuraminidase (NA) genes code for the major virus surface glycoproteins [2]. Based on their antigenic properties, AIVs are classified into different subtypes. Sixteen subtypes of HA (H1-H16) and nine of NA (N1-N9) have been identified in birds [3], in different combinations. Two HA (H17 and H18) and two NA (N10 and N11) were identified exclusively in bats [4].

The AIV H9N2 was first detected in the USA, in 1966 [5], but now, it is considered as a worldwide distributed pathogen [6]. The H9N2 AIV subtype harbors two phylogenetic lineages: the North American lineage and the Eurasian lineage [1, 7]. The last one contains different prototypes that are grouped in at last three sub-lineages. The G1-like sub-lineage is represented by the prototype virus A/Quail/Hong Kong/G1/97; the Y280-like sub-lineage by A/Duck/Hong Kong/Y280/97, A/Chicken/Beijing/1/94, and A/Chicken/Hong Kong /G9/97; and finally, the Korean-like sub-lineage by A/Chicken/Korea/38349-p96323/96 and A/Duck/Hong Kong/Y439/97 [7].

Despite their low pathogenicity, AIV H9N2 viruses have led to heavy economic losses, particularly during coinfections with other respiratory pathogens [8, 9]. Also, it has been reported that low pathogenic avian influenza virus (LPAIV) H9N2 can easily undergo genetic reassortment and donate internal gene segments to highly pathogenic avian influenza viruses (HPAIV) H5 and H7 [6, 10–12]. In addition, different studies, showed that circulating H9N2 strains have acquired affinity to mammalian like-receptors and gained high virulence and pathogenicity through substitutions in their viral proteins [13, 14]; the most known substitutions are in the HA protein that promotes virus binding to cellular receptors. Likewise, the polymerase complex protein, which is composed of three subunits (polymerase basic 2 “PB2” protein, polymerase basic 1 “PB1” and polymerase acidic “PA” proteins), harbors multiple molecular markers, which also affect host tropism and pathogenicity of influenza viruses. Similarly, the matrix (M) and the non-structural (NS) proteins also contain amino acids (aa) that contribute to the virus growth capabilities in mammalian cell cultures [15].

Currently, very limited data is available on the circulation of AIVs in Algeria. Concerning HPAIVs, the Algerian veterinary services have reported, in November 2016, the first case of H7N1 infection in the natural park of Ghardaia, in the South of Algeria. The collected samples from dead migratory birds tested positive by specific quantitative RT-PCR. However, no control measures were applied [16], with the concern of spread of virus from migratory birds to domestic poultry. Recently, Jeevan et al., (2019) have reported isolation of H9N2 AIV from an outbreak observed, in 2017, in the Central region of Algeria. Their study focused on phylogenetic characterization and pathogenicity of the isolates in experimental animals [17]. However, the full-length genome sequence analysis had not been carried out. In the present study, we report the isolation and the full-length genome sequencing of 10 AIV-H9N2, isolated from broiler poultry farms, in the North East of Algeria (province of Batna), during 2017. Genome sequences were successfully obtained for the all isolate genomes, followed by phylogenetic and detailed amino acid (aa) sequence analyses of determinants of virulence, host specificity and pathogenicity.

## Materials and methods

### Case history

In the third week of April 2017, an epizootic outbreak of a fatal and highly contagious respiratory disease was reported in broiler flocks in the province of Batna (North East of Algeria). The mortality rate in the affected farms was variable, ranging from 40 to 60%, with an average daily mortality of 300 chickens per flock. Outbreaks particularly occurred in flocks older than 45 days of age (age of slaughter in Algeria: > 50 days). This epizootic outbreak subsequently spread very quickly to all broiler farms in the province of Batna. Neighboring provinces (Sétif and M’sila) were also affected. At necropsy, the most observed lesions were mainly localized in the respiratory and digestive tracts, such as hemorrhages in the trachea and intestines. Yellow fibrinous exudates were also found in the lumen of the trachea, with fibrinous casts in bronchi.

### Sampling

Given the constraints encountered in the field, 10 broiler flocks affected when the outbreak was declared were sampled according to their accessibility. Twenty organ pools (trachea, kidneys, liver, and intestines) were collected from April to September 2017 (two organ pools per flock); each organ pool containing samples (trachea, kidneys, liver or intestines) collected from five randomly selected broiler chickens. Samples were immediately placed in cryotubes containing viral transport medium (PBS-glycerol, Sigma®, antibiotics and antifungi), and then transported to the laboratory at 4 °C, and stored at − 80 °C until used.

### RNA extraction

Each organ pool was homogenized in DMEM (Dulbecco’s Modified Eagle Medium) (Gibco, Thermo Fisher scientific) supplemented with 1% penicillin-streptomycin, and then clarified by centrifugation (10,000 rpm for 15 min at + 4 °C). The upper aqueous phase was collected for RNA extraction. Total RNA was extracted by the Trizol method (Invitrogen, Carlsbad, CA, USA), according to the manufacturer’s instruction.

### AIV detection and sub-typing

Avian influenza A virus was detected by real time RT-PCR, using Agpath One-Step kit (Ambion, applied Biosystems, Grand Island, New York) with primers and probes specific to the conserved region of the matrix (M) gene, previously described by Spackman et al., (2002) [18]. Reactions were performed according to the following protocol: one cycle of 45 °C for 10 min and 95 °C for 10 min, followed by 40 cycles of 95 °C for 30 s and 60 °C for 1 min. Positive samples were then subtyped by RT-PCR, using AIV H9 detection kit (Intron Biotechnologie®, Korea), using the following protocol: 45 °C for 30 min and 94 °C for 5 min, followed by 40 cycles of 94 °C for 30 s, 58 °C for 30 s, 72 °C for 40 s, and a final elongation step of 72 °C for 5 min.

### Virus isolation and characterization

The upper aqueous phases from organ pools that were AIV positive, were used to isolate the virus in 9-day-old specific pathogen free (SPF) embryonated chicken eggs (PFIE, INRAE, Nouzilly, France), inoculated via the allantoic route. Embryonic mortality within 24 h after inoculation was considered nonspecific. The allantoic fluid was then harvested 48 h post-inoculation and total viral RNA was extracted using the QIAamp® Viral RNA Mini Kit (Qiagen®, Germany), following the manufacturer’s instructions.

### Full genome amplification

The full-length genome amplification of the studied strains was carried out by conventional RT-PCR using segment specific primers, as previously described by Hoffmann et al., (2001) [19]. The RT-PCR reaction was performed in a Peqlab PeqSTAR thermocycler (VWR), with the following program: 50 °C for 30 min, 95 °C for 15 min, followed by 40 cycles of 95 °C for 30 s, 57 °C for 30 s and 72 °C for 2.5 min. A final extension step at 72 °C for 10 min was then performed.

### Sequencing and phylogenetic analyses

The PCR products of the expected length were purified from the agarose gel using the QIAquick® Gel Extraction Kit (Qiagen®, Germany), following the manufacturer’s instructions. The purified amplicons were subjected to nucleotide sequencing using the same primers as in RT-PCR by the GATC Biotech Platform (Germany). Consensus DNA sequences were constructed from forward and reverse sequences in a DNA Baser v4.12.0 [20] . Nucleotide (nt) and aa sequence alignments were carried out using ClustalW in BioEdit Sequence Alignment Editor v7.2.5. The Blast software programs (https://blast.ncbi.nlm.nih.gov/Blast.cgi) were used to determine the sequence similarity of the Algerians strains. Phylogenetic trees were constructed by Maximum likelihood method in a MEGA X 10.1.8 program [21], using the General Time Reversible (GTR) nucleotide substitution model; one thousand bootstrapping replicates being used to estimate the robustness of the tree branches. The nucleotides sequences of all studied strains are available in GenBank database, under accession numbers summarized in Table 1.

**Table 1:** Accession numbers of gene sequences of studied Algerians H9N2

Genes	Strains									
	6BBD	8BBD	9BBD	10BBD	12BBD	13BBD	15BBD	17BBD	18BBD	19BBD
PB2	MK796878	MK796879	MK796880	MK796881	MK796882	MK796883	MK796884	MK796885	MK796886	MK796887
PB1	MK796868	MK796869	MK796870	MK796871	MK796872	MK796873	MK796874	MK796875	MK796876	MK796877
PA	MK796835	MK796836	MK796837	MK796838	MK796839	MK796840	MK796841	MK796842	MK796843	MK796844
HA	MK788274	MK788275	MK788276	MK788277	MK788278	MK788279	MK788280	MK788281	MK788282	MK788283
NP	MK796798	MK796799	MK796800	MK796801	MK796802	MK796803	MK796804	MK796805	MK796806	MK796807
NA	MK796787	MK796788	MK796789	MK796790	MK796791	MK796792	MK796793	MK796794	MK796795	MK796796
M	MK803312	MK803313	MK803314	MK803315	MK803316	MK803317	MK803318	MK803319	MK803320	MK803321
NS	MK796816	MK796817	MK796818	MK796819	MK796820	MK796821	MK796822	MK796823	MK796824	MK796825

6BDD, 8BDD, 9BDD, 10BDD, 12BDD, 15BDD, 17BDD, 18BDD and 19BDD are used respectively for A/Chicken/Algeria/6BBD/2017, A/Chicken/Algeria/8BBD/2017, A/Chicken/Algeria/9BBD/2017, A/Chicken/Algeria/10BBD/2017, A/Chicken/Algeria/12BBD/2017, A/Chicken/Algeria/13BBD/2017, A/Chicken/Algeria/15BBD/2017, A/Chicken/Algeria/17BBD/2017, A/Chicken/Algeria/18BBD/2017 and A/Chicken/Algeria/19BBD/2017

Phylogenic characterization of the studied Algerians strains was performed for all their AIV gene segments, in comparison with selected AIV H9N2 strains from Genbank. For amino acid analyses, selected sequences were subjected to ClustalW multiple sequence alignment [22] . The analysis was performed for different molecular markers of host viral tropism, pathogenicity and virulence, as previously described for LPAIV H9N2 and other subtypes.

## Results

### AIV detection

All 20 organ pools tested to detect AIV genome were positive to AIV H9N2 subtype. Virus from 10 of the 20 pools could successfully be isolated in embryonnated eggs and characterized.

### Phylogenetic analysis of the surface genes

**Fig. 1:**
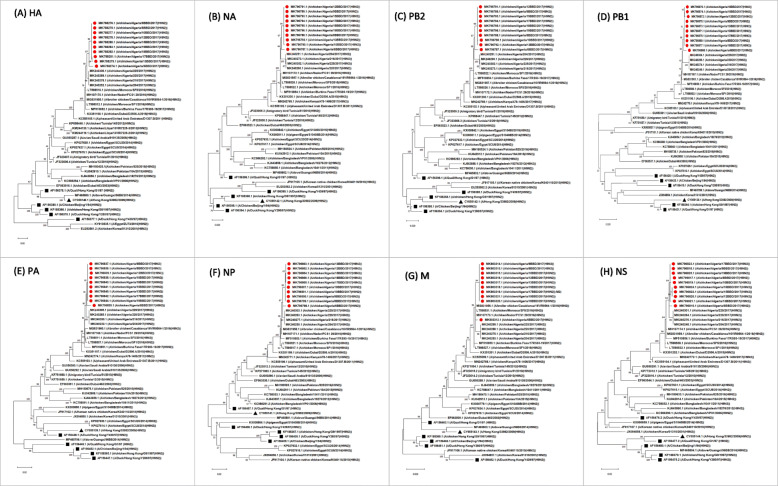
Phylogenetic trees of the gene segments HA, NA, PB2, PB1, PA, NP, M, NS (from A-H respectively), showing the evolutionary relationship between the studied isolates (red circles) and the reference AIVs from GenBank (black squares) and the WHO recommended vaccine strains (black triangles). The trees were obtained using the maximum likelihood (ML) method, using the general time reversible (GTR) nucleotide substitution model, with the MEGA X, version 10.1.8. Numbers at the nodes indicate the bootstrap values (1000 replicates)

Phylogenetic analysis showed that HA and NA genes of all studied AIV H9N2 grouped in the G1 sub-lineage of the Eurasian lineage. The highest phylogenetic relationship and nucleotide identity were shown with the prototype A/quail/Hong Kong/G1/97 rather than other AIV H9N2 prototypes such as A/Duck/Hong Kong/Y280/97 and A/Chicken/Hong Kong/G9/1997. All Algerian strains were also related to each other (nucleotides identity ranging from 99.98 to 100% and from 99.01 to 100% for the HA and NA, respectively) and grouped together in the phylogenic tree (bootstrap value: 99 for the HA and NA,) (Fig. 1). Besides, the isolate A/Chicken/Algeria/6BBD/2017 exhibited the lowest nucleotide identity, as compared to all studied H9N2 strains. Our strains were also monophyletic with recently published Algerians AIV H9N2 sequences, such as A/Chicken/Algeria/216/2017 and A/Chicken/Algeria/216/2017, isolated in the central region of Algeria in 2017 (Fig. 1) with high nucleotide sequence identities (99% for HA and 98 to 99% for NA).

The Algerian H9N2 studied clustered with AIV H9N2 from different geographic areas such as Morocco (more than 99% genetic identity) and the United Arab Emirates (UAE) (more than 98% identity), belonging to the G1 Middle Eastern group. Our isolates were also highly similar to AIV H9N2 isolated, in 2017 in Burkina Faso (more than 98% identity for both HA and NA). On the other hand, the current Algerian AIV H9N2 were relatively distant from the Tunisian (92% for the HA and NA) and the Libyan strains (91% nt identity for HA) isolated in 2010 and 2013, respectively.

### Phylogenetic analysis of internal genes

As for surface genes, all internal genes (PB2, PB1, PA, NP, M and NS) of the studied H9N2 isolates were compared to their counterparts from GenBank to establish phylogenetic trees and genetic similarity matrixes at the nucleotide level. Here again, the internal genes of the Algerian strains clustered within the G1 sub-lineage, and were closely related to each other, as shown both by their high nucleotide identities (99.30–99.96%, 99.43–100%, 99.30–100%, 90.22%-100, 98.99 - 99.90%, and 99.29 -100% for PB2, PB1, PA, NP, M, and NS gene segments, respectively) and the bootstrap values (Fig. 1). No reassortment event was observed. The strain A/Chicken/6BDD/Algeria/2017 remained the most distinct strain. In addition, the studied strains showed high nucleotide sequence identity with those isolated during the same year (2017), with more than 98, 98 to 99%, 98 to 99%, more than 99, 98, and 99% identity for PB2, PB1, PA, NP, M, and NS, respectively.

As compared to AIV H9N2 isolated in other countries, the highest nucleotide identities and phylogenic relationship were shared with those reported in Morocco, Burkina Faso and UAE, already grouped within the G1 sub-lineage (Fig. 1). The results of the nucleotide sequence comparison (percent identity) between the genes segments (PB2, PB1, PA, HA, NP, NA, M and NS) of the studied Algerian H9N2 strains and selective isolates are illustrated in Table 2. Given the highest nucleotide identity found between the 10 studied isolates as well as compared to selective used strains, only three isolates, A/Chicken/19BDD/Algeria/2017, A/Chicken/9BDD/Algeria/2017 and the most distinct A/Chicken/6BDD/Algeria/2017 isolate were presented in Table 2.

**Table 2:** Nucleotide sequence comparison between the genes segments of the studied Algerian H9N2 strains and selective isolates (percent identity, %)

Algerian strains	Genes	References strains					Contemporary Algerian’s strains		Strains from neighboring country				UAE
		HK-G1	HK-Y280	HK-Y439	CK-Bei	HK-G9	Alg-216	Alg-225	Morocco-SF1	Morocco-SF5	Tun-12	Tin-51	Dubai-D2506.A
19BDD	**PB2**	86.77	84.75	85.79	84.89	88.18	98.90	98.95	99.12	99.08	93.16	93.16	98.59
	**PB1**	88.13	87.06	88.54	88.60	88.10	99.08	99.08	99.47	99.52	94.17	94.17	98.33
	**PA**	88.76	87.14	91.80	87.65	86.81	99.30	99.35	99.35	99.44	94.61	94.61	98.74
	**HA**	89.14	86.64	81.75	87.57	87.35	98.92	98.80	99.16	98.92	92.53	92.53	98.62
	**NP**	94.33	87.50	91.10	87.81	88.43	99.13	99.27	99.20	99.13	95.32	95.32	97.86
	**NA**	89.68	87.49	84.08	89.05	87.46	98.37	98.37	99.22	99.36	92.20	92.35	98.44
	**M**	94.16	92.86	89.50	93.02	93.24	98.79	98.79	99.39	99.28	96.23	96.23	98.35
	**NS**	88.70	88.94	91.47	89.54	89.23	99.41	99.41	99.05	99.28	95.15	95.15	98.58
9BDD	**PB2**	86.82	84.75	85.79	84.89	87.37	98.95	98.99	99.16	99.12	93.20	93.20	98.63
	**PB 1**	88.04	87.97	88.45	88.51	88.10	99.08	99.08	99.47	99.52	94.17	94.17	98.33
	**PA**	88.76	87.14	91.80	87.65	86.81	99.30	99.35	99.35	99.44	94.61	94.61	98.74
	**HA**	89.14	86.64	81.75	87.57	87.35	98.92	98.80	99.16	98.92	92.53	92.53	98.62
	**NP**	94.33	87.50	91.10	87.81	88.43	99.13	99.27	99.20	99.13	95.32	95.32	97.86
	**NA**	89.68	87.49	84.08	89.05	87.46	98.37	98.37	99.22	99.36	92.20	92.35	98.44
	**M**	94.16	92.87	89.51	93.02	93.35	98.89	98.89	99.39	99.28	96.23	96.23	98.35
	**NS**	88.70	88.94	91.47	89.54	89.23	99.41	99.41	99.05	99.28	95.15	95.15	98.58
6BDD	**PB2**	87.04	85.06	86.10	85.20	87.59	99.04	99.08	99.25	99.21	93.29	93.29	98.68
	**PB 1**	88.13	88.06	88.54	88.60	88.18	98.81	98.81	99.21	99.25	93.94	93.94	98.07
	**PA**	88.39	86.82	91.52	87.32	86.49	98.88	98.93	98.93	99.02	94.19	94.19	98.33
	**HA**	89.15	86.71	82.07	87.70	87.40	98.50	98.38	98.86	98.62	92.30	92.29	98.32
	**NP**	94.19	87.50	90.98	87.74	88.31	99.13	99.26	99.20	99.13	95.12	95.12	97.86
	**NA**	89.46	87.14	83.94	88.77	87.31	98.23	98.23	99.08	99.22	92.06	92.20	98.30
	**M**	94.16	92.67	89.61	92.81	93.25	98.99	98.99	99.59	99.49	96.44	96.44	98.55
	**NS**	88.46	88.70	91.47	89.30	88.99	99.17	99.17	98.81	99.04	94.91	94.91	98.34

HK-G1: A/Quail/Hong Kong/G1/97. HK-Y280: A/Duck/Hong Kong/Y280/97. HK-Y439: A/duck/Hong Kong/Y439/97. CK-Bei: A/Chicken/ Beijing/1/94. HK-G9: A/Chicken/Hong Kong/G9/1997. Alg-216: A/Chicken/Algeria/216/2017. Alg-225: A/Chicken/Algeria/225/2017. Morocco-SF1: A/Chicken/Morocco/SF1/2016. Morocco-SF5: A/Chicken/Morocco/SF5/2016. Tun-12: A/Chicken/Tunisia/12/2010. Tun-51: A/MigratoryBird/Tunisia/51/2010. UAE: United Arab Emirates. Dubai-D2506.A: A/Chicken/Dubai/D2506.A/2015

### Molecular characterization

Molecular markers known in the literature for their roles in pathogenesis, host tropism, enhanced replication or drug resistance have been carefully studied and are summarized in Tables 3, 4, and 5 (for HA, NA, and main internal proteins). Of interest, the current Algerians AIV H9N2 share a monobasic cleavage site motif ^335^RSSR/GLF^341^ (H9 numbering) (Table 3), which is similar to that of the prototype A/Quail/Hong Kong/G1/97 strain, defined as LPAIV in poultry. Our viruses harbor HA markers of human-like α2,6 sialic acids binding preference (A198T, Q234L and Q235I). In addition, a new substitution (T197A), with unknown biological function and no previous report for H9N2 viruses, was revealed in three out the 10 Algerian strains. All studied isolates shared seven potential glycosylation sites (PGS) at the same position: 29–31 (NTS), 105–107 (NGT), 141–143 (NVT), 298–300 (NST), 305–307 (NIS), 492–494 (NGT) and 551–553 (NGS). The PB1-F2 protein of Algerian isolates was truncated at aa 52. Additionally, all Algerian strains harbored the S31N substitution in M2 protein, which is associated with drug resistance. None of the known neuraminidase inhibitors resistance markers was detected in NA proteins. The NS protein showed the “GSEV” PDZ ligand (PL) C-terminal motif and no 80–84 deletion.

**Table 3:** Amino acid sequences analysis of the HA protein of Algerian isolates

Avian influenza viruses	Receptor binding Site (RBS)											Right-edge of binding pocket					Left-edge of binding pocket					Connecting peptide aa sequence						Potentials glycosylation sites (PGS)
	106	110	161	163	166	191	198	202	203	234	235	148–152 ^a^142–146					232–236 ^b^233–237					333–338 ^c^366–441						
A/Quail/Hong_Kong/G1/97	V	P	W	T	S	H	E	L	Y	L	Q	S	R	A	C	S	N	D	L	Q	G	P	A	R	S	S	R	8
A/Turkey/Wisconsin/1/1966	–	–	–	–	–	–	–	–	–	Q	–	–	–	–	–	–		G	Q			–	–	V	–	–		7
A/Duck/Hong_Kong/Y280/97	–	–	–	–	N	N	T	–	–	–	–	–	K	–	–	–	–	G	–	–	–	–	–	–	–	–	–	6
A/Duck/Hong_Kong/Y439/97	–	–	–	–	–	–	–	–	–	Q	–	–	–	–	–	–			Q			–	–	A	–	N	–	6
A/Guangdong/MZ058/2016 *	–	–	–	–	D	N	T	–	–	–	M	–	T	–	–	–	–	G	–	M	–	–	S	–	–	–	–	7
A/Lebanon/11 L046/2012 (H3N2)	–	–	–	–	N	–	D	–	–	I	P	–	–	R	S	N	R	N	I	P	S	–	E	K	Q	T	–	12
A/Minnesota/19/2011(H1N2)	–	–	–	–	N	–	N	–	–	Q	E	–	A	S	–	–	R	–	Q	E	–	–	S	I	Q	–	–	9
A/Paris/650/2004 (H1N1)	–	–	–	–	N	–	D	–	–	Q	E	–	A	S	–	–	R	–	Q	E	–	–	S	I	Q	–	–	10
A/Egypt/N02752/2009(H5N1)	–	–	–	–	D	–	–	–	–	Q	S	–	S	–	–	P	–	G	Q	S	–	–	Q	G	E	R	R	7
A/Shanghai/02/2013(H7N9)	I	–	–	–	L	–	–	–	–	L	S	T	S	–	–	R	–	G	–	S	–	–	I	P	K	G	–	5
A/Chicken/Karachi/NARC100/2004 (H7N3)	I	K	C	R	G	S	G	I	I	Q	Q	^a^G	A	T	S	S	^b^N	G	Q	S	G	^c^P	k	R	R	K	R	2
A/Chicken/Morocco/SF1/2016	–	–	–	–	N	–	A	–	–	**–**	I	–	K	S	–	–	–	G	–	I	–	H	–	–	–	–	–	7
A/Chicken/Tunisia/12/2010	–	–	–	–	N	–	A	–	–	**–**	I	–	K	S	–	–	–	G	–	I	–	–	–	–	–	–	–	7
A/Chicken/Egypt/F12168B/2016	–	–	–	–	N	–	A	–	–	**–**	I	–	K	S	–	–	–	G	–	I	–	–	–	–	–	–	–	7
A/Chicken/Dubai/D2506.A/2015		–	–	–	N	–	A	–	–	**–**	I	–	K	S	–	–	–	G	–	I	–	H	–	–	–	–	–	7
A/Chicken/Pakistan/10A/2015	–	–	–	–	N	–	A	–	–	**–**	I	–	K	S	–	–	–	G	–	I	–	–	–	K	–	–	–	7
A/Chicken/B Faso/17RS93–9/2017	–	–	–	–	N	–	A	–	–	**–**	I	–	K	S	–	–	–	G	–	I	–	H	–	–	–	–	–	8
A/Chicken/Algeria/6BBD/2017	–	–	–	–	N	–	T	–	–	–	I	–	K	S	–	–	–	G	–	I	–	H	–	–	–	–	–	7
A/Chicken/Algeria/8BBD/2017	–	–	–	–	N	–	T	–	–	**–**	I	–	K	S	–	–	–	G	–	I	–	H	–	–	–	–	–	7
A/Chicken/Algeria/9BBD/2017	–	–	–	–	N	–	T	–	–	**–**	I	–	K	S	–	–	–	G	–	I	–	H	–	–	–	–	–	7
A/Chicken/Algeria/10BBD/2017	–	–	–	–	N	–	T	–	–	**–**	I	–	K	S	–	–	–	G	–	I	–	H	–	–	–	–	–	7
A/Chicken/Algeria/12BBD/2017	–	–	–	–	N	–	T	–	–	**–**	I	–	K	S	–	–	–	G	–	I	–	H	–	–	–	–	–	7
A/Chicken/Algeria13BBD/2017	–	–	–	–	N	–	T	–	–	**–**	I	–	K	S	–	–	–	G	–	I	–	H	–	–	–	–	–	8
A/Chicken/Algeria/15BBD/2017	–	–	–	–	N	–	T	–	–	**–**	I	–	K	S	–	–	–	G	–	I	–	H	–	–	–	–	–	7
A/Chicken/Algeria/17BBD/2017	–	–	–	–	N	–	T	–	–	**–**	I	–	K	S	–	–	–	G	–	I	–	H	–	–	–	–	–	8
A/Chicken/Algeria/18BBD/2017	–	–	–	–	N	–	T	–	–	**–**	I	–	K	S	–	–	–	G	–	I	–	H	–	–	–	–	–	7
A/Chicken/Algeria/19BBD/2017	–	–	–	–	N	–	T	–	–	**–**	I	–	K	S	–	–	–	G	–	I	–	H	–	–	–	–	–	7

Strains whose subtype is not indicated in parentheses are H9N2; * Human H9N2 viruses; Dash indicates that the amino acid is similar to that in the strain A/Quail/Hong Kong/G1/97 at the same position; H9, H7, and H5 numbering for H9N2, H7, and H5N1, respectively. ^a, b, c^ letters indicates aa respectively at positions 142–146, 233–237 and 366–441 of the right-edge of binding pocket, the left-edge of binding pocket and connecting peptide of Karachi H7N3 virus

**Table 4:** Amino acid sequences analyses of NA protein of Algerian isolates

Avian influenza viruses	Hemadsorbing site (HB)																	Potentials glycosylation sites (PGS)
	1st loop 366–373								2nd loop 399–404					3rd loop 431–433				
A/Quail/Hong_Kong/G1/97	I	K	K	D	S	R	S	G	D	S	D	I	R	S	P	Q	E	6
A/Turkey/Wisconsin/1/1966	–	S	–	–	–	–	–	–	–	–	N	N	W	–	–	–	–	7
A/Duck/Hong_Kong/Y280/97	–	–	E	–	–	–	–	–	–	–	–	N	W	–	–	–	–	7
A/Duck/Hong_Kong/Y439/97	–	S	–	–	–	–	–	–	–	N	N	N	W	–	–	–	–	10
A/Guangdong/MZ058/2016^a^	–	–	N	G	–	–	–	–	–	–	–	D	W	–	–	–	–	8
A/Lebanon/11 L046/2012 (H3N2)	–	N	E	T	–	–	L	–	–	R	G	D	–	–	K	E	–	8
**A/Minnesota/19/2011(H1N2)**	–	N	E	T	L	–	L	–	E	K	G	D	–	–	K	E	–	9
A/Paris/650/2004 (H1N1)	K	S	N	R	L		K	–	I	T	–	D	W	–	–	R	–	9
**A/Egypt/N02752/2009 (H5N1)**	K	S	T	N	–	–	–	–	I	T	–	D	W	–	–	K	–	3
**A/Shanghai/02/2013 (H7N9)**	–	S	T	A	–	–	–	–	L	N	A	D	W	–	–	K	–	7
**A/Chicken/Karachi/NARC-100/2004 (H7N3)**	S	N	S	G	R	S	G	F	N	N	D	W	S	G	N	K	N	6
A/Chicken/Morocco/SF1/2016	–	–	–	–	L	–	A	–	–	N	–	N	W	–	–	R	–	8
A/Chicken/Tunisia/12/2010	–	–	–	–	L	–	A	–	–	–	–	N	W	–	–	–	–	7
**A/Chicken/Egypt/F12168B/2016**	–	–	–	–	–	–	A	–	–	–	–	S	W	–	–	–	–	7
**A/Chicken/Dubai/D2506.A/2015**	–	–	–	–	L	–	A	–	–	–	–	N	W	–	–	R	–	8
**A/Chicken/Pakistan/10A/2015**	–	E	–	–	–	–	T	–	–	–	G	N	–	–	–	–	–	7
**A/Chicken/B Faso/17RS93–19/2017**	–	–	–	–	L	–	A	–	–	N	–	N	W	–	–	R	–	8
**A/Chicken/Algeria/6BBD/2017**	–	–	–	–	L	–	A	–	–	N	–	N	W	–	–	–	–	8
**A/Chicken/Algeria/8BBD/2017**	–	–	–	–	L	–	A	–	–	N	–	N	W	–	–	R	–	8
A/Chicken/Algeria/9BBD/2017	–	–	–	–	L	–	A	–	–	N	–	N	W	–	–	R	–	8
**A/Chicken/Algeria/10BBD/2017**	–	–	–	–	L	–	A	–	–	N	–	N	W	–	–	R	–	9
A/Chicken/Algeria/12BBD/2017	–	–	–	–	L	–	A	–	–	N	–	N	W	–	–	R	–	8
A/Chicken/Algeria/13BBD/2017	–	–	–	–	L	–	A	–	–	N	–	N	W	–	–	R	–	8
A/Chicken/Algeria/15BBD/2017	–	–	–	–	L	–	A	–	–	N	–	N	W	–	–	R	–	8
A/Chicken/Algeria/17BBD/2017	–	–	–	–	L	–	A	–	–	N	–	N	W	–	–	R	–	8
A/Chicken/Algeria/18BBD/2017	–	–	–	–	L	–	A	–	–	N	–	N	W	–	–	R	–	8
A/Chicken/Algeria/19BBD/2017	–	–	–	–	L	–	A	–	–	N	–	N	W	–	–	R	–	8

^a^Human H9N2 viruses**;** Dash indicates that the aa is similar to that in the strain A/Quail/Hong Kong/G1/97 at the same position

**Table 5:** Analysis of amino acid sequences of PB2, PB1, PA, NP, M1, M2, M1, and NS2 of Algerians AIV H9N2 viruses in comparison with references strains

Genes	Molecular determinants of virulence				Molecular determinants of host specificity and host pathogenicity				Molecular determinants of drug resistance			Miscellaneous
	Sites	ALG	HK-G1	Virulent	Sites	ALG	HK-G1	Mammalian preference	sites	ALG	HK-G1	
PB2	89	V	V	L	44	A	A	S				
	147	V	M	L	64	M	M	T				
	250	V	V	G	81	T	T	M				
	253	D	D	N	158	E	E	G				
	404	F	F	L	190	K	R	K				
	504	V	V	V	199	A	A	S				
					271	T	T	A				
					318	R	K	R				
					339	K	K	T				
					526	K	K	R				
					543	E	E	D				
					588	A	A	V				
					590	S	S					
					591	Q	Q	K				
					627	E	E	K				
					643	S	S	T				
					655	V	A	V				
					661	T	T	A				
					666	T	T	I				
					701	D	D	N				
					702	Q	K	R				
					714	S	S	R				
PB1	105	N	N	S	13	P	P	P				
	317	M	I	I	207	K	K	R				
	577	K	K	E	336	V	V	I				
	622	G	G	G	375	N	N	S				
					436	Y	Y	H				
					578	K	K	Q				
					677	T	T	M				
PB1-F2												Truncated protein of 52 aa
PA	127	V	V	V	28	P	P	L				
	550	L	L	L	44	V	V	I				
	672	L	L	L	55	D	D	N				
					57	R	R	Q				
					63	V	V	I				
					97	T	T	I				
					100	I	V	A				
					133	E	E	G				
					142	K	K	N/E				
					225	S	S	C				
					241	C	C	Y				
					268	L	L	I				
					312	R	K	R				
					343	A	A	S				
					347	D	D	E				
					356	K	K	R				
					382	E	E	D				
					383	D	D	D				
					400	T	L	L				
					404	A	A	S				
					409	N	S	N				
					515	T	T	T				
					552	T	T	S				
					556	Q	Q	R				
					573	I	I	V				
					615	K	R	N				
PA-X												Full length 252 aa
NP	210	D	D	D	31	R	R	K				
					33	V	V	I				
					34	G	G	N				
					61	I	I	L				
					100	R	R	V				
					109	I	I	V				
					127	E	E	D				
					136	M	M	M				
					214	R	R	K				
					283	L	L	P				
					293	R	R	K				
					305	R	R	K				
					313	F	F	Y				
					357	Q	Q	K				
					372	D	E	D				
					375	D	D	G/E				
					398	Q	Q	Q				
					422	R	R	K				
					430	K	K					
					442	T	T	A				
					455	D	D	E				
					480	D	D					
M1	30	D	D	D	15	I	I	I				
	43	M	M	M	115	V	R	I				
	215	A	A	A	121	T	T	A				
					137	T	T	A				
M2	64	S	S	S/A/F	11	T	T	I	26	L	L	
	69	P	P	P	16	D	G	G/D	27	V	V	
					28	V	V	I/V	31	N	S	
					55	F	F	F	34	G	G	
					57	Y	Y	H	38	L	L	
					86	V	V	A				
NS1	42	**S**	S	S	227	G	E	K/R				Non 80TIASV84 deletion GSEV PL motif
	92	D	E	E								
	103	L	L	F								
	106	M	I	M								
	149	A	A	A								
	189	D	D	N								
NS2	31	M	M	I								
	56	H	H	Y								

*ALG* currents Algerians AIV H9N2, *HK-G1* A/quail/Hong Kong/G1/97

## Discussion

AIV H9N2 has a widespread distribution. In North Africa, the virus was detected in Tunisia [13] and recently in Morocco [14]. However, no official report is available on the circulation of H9N2 viruses in Algeria, except for the recent declaration of one case in the North Center of the country [17]. Besides, in the province of Batna (Northeast Algeria), an outbreak of highly contagious respiratory disease was reported in broiler flocks, in 2017 and we showed here that the outbreak was due to H9N2 AIV. A high mortality rate (40 to 60%) was recorded in unvaccinated broilers; such high mortality rate contrasted with a low pathogenic nature of isolated H9N2 subtype and were certainly due to co-infections of H9N2 AIV with other respiratory pathogens particularly infectious bronchitis virus (IBV), *Escherichia coli*, HPAIV, *Mycoplasma gallisepticum* (MS), *Ornithabacterium rhinotracheale*, avian metapneumoviruses (aMPV) and Newcastle diseases virus (NDV) [9, 23–26].

The full-length genome sequencing of AIV viruses allowed molecular characterization and more knowledge about the molecular genetic markers of virulence and pathogenicity.

### Phylogenetic analyses

Phylogenetic analyses of HA and NA surface glycoproteins of the currents H9N2 isolated strains showed a same evolutionary pattern. All Algerians H9 and N2 gene sequences were monophyletic and grouped with different AIV H9N2 strains from North Africa and the Middle East, in the G1-like AIV’s group. These results are not surprising given the large spread of the G1-like group in the Middle East, which has facilitated transmission of the disease in North Africa and neighboring countries. Different countries sharing borders with Algeria were also infected by the same lineage, such as Morocco [14], Tunisia [13] and Libya [27]. In the Middle East, Egypt was also infected with AIV H9N2 of the G1 lineage [28].

The monophyletic aspect shared with AIV H9N2 viruses from Morocco, United Arab Emirates, Burkina Faso and Saudi Arabia suggests virus spread in the region. The high nucleotide identities and the phylogenetic relationship between the Algerian and the Moroccan AIV H9N2 isolates were not surprising and suggest that these isolates have a common origin [29]. The reinforcement of biosecurity measures and the study of the factors of introduction and propagation of AIV H9N2 in industrial breeding farms are crucial to reduce both the prevalence of the disease and the high economic losses. On the other hand, the low nucleotide sequence identity observed between the Algerian and the Tunisian strains, isolated in 2017 and 2010–2012, respectively, showed that temporal distance plays a role on virus evolution here, and suggests a surveillance gap in the 2012–2017 period in the region.

Phylogenetic analyses also showed that all internal genes (PB2, PB1, PA, NP, M, and NS) of the Algerians H9N2 strains are homogeneous and grouped with those from neighboring countries (Morocco and Tunisia) and more globally from the Middle East, under the G1 lineage. Despite the reassortment observed among different AIV subtypes (H9, H5, H7) [30] and lineages, our findings suggest that the Algerians H9N2 viruses did not undergo genetic reassortment with other AIV subtypes and lineages.

### Molecular characterization of surface glycoproteins

The Algerians strains shared the ^335^RSSR/GLF341 (H9 numbering) sequence motif at their HA1-HA2 connecting peptides, which indicates their low pathogenicity [31]. It has been reported that this motif is characteristic of LPAIV H9N2 viruses from Middle East [31, 32] and Asia [32] and well adapted to poultry [33]. However, some studies have reported that H9N2 viruses may undergo punctual substitutions at the HA1/HA2 connecting peptide sequence and acquire the dibasic pattern K-S-S-R at the cleavage site [34], thus increasing their pathogenicity [13, 35].

The aa residues 183/191, 190/198, 226/234, 227/235 and 228/236 (H3/H9 numbering) at the RBS of the HA protein are key determinants [34, 36]. Residue position 226 is the most crucial for host specificity. Thereby, AIV having the signature Q226, preferentially bind avian host receptor via α [2, 3] Gal-type binding [37]. The current Algerian strains harbored two aa substitutions, N191H, Q234L, within the RBS, which suggests they may be able to infect other species than birds, such as mammalian hosts. Particularly, the substitution Q234L enhancing the affinity shift from avian toward human-like receptors [37]. In addition, Matrosovich et al., (2008) suggested that the residue 234 L is typical of human pandemic AIV H2 and H3 subtypes, and is not characteristic of AIV [38]. Similarly, the residue 191H was associated with a binding preference for human receptors on the respiratory epithelial cells [28], direct contact transmission of AIV H9N2 in ferrets [39] and more efficient replication in human airway cell cultures [40].

Two additional substitutions, A198T and Q235I, on the RBS, were observed in the Algerian H9N2 strains. The first substitution is also present in A/duck/Hong Kong/Y280/97, the prototype of Y280-like viruses, and A/Guangdong/MZ058/2016, a human H9N2 virus. Their functions remain unknown [33, 34, 36]; however, Matrosovich et al., (2001), have reported that residue 190/198 (H3/H9 numbering) influences α [2–6] receptor binding (human-like receptor) and consequently mammalian infections [41]. At the same positions, different substitutions were previously described, such as 190 T/V/A (H3 numbering) [42]. Therefore, 198 T may allow binding of the Algerian strains to α [2–6] human-like receptors, but with lower affinity as compared to residues A and V at the same position, 198 V characteristic of HPAIV, conferring the most affinity binding towards human receptors [41]. Considering the combination of multiple substitutions in the RBS, the Algerian strains may have higher binding affinity to human-like receptors.

The Blast analysis (NCBI) of the HA protein showed an exclusive mutation T197A for our strains in comparison with H9N2 subtype viruses published in GenBank; the strains isolated the same year in the Center of Algeria, did not harbor this mutation. Interestingly, this mutation was conserved in all human H1N1 strains of 2014–2015, in India [43] and in Italian 2009 strains [44]. The only H9N2 strain that displays a substitution at the same position (T197D) was isolated from a 2-year-old child in Changsha City, in China [45]. Biological impact of such mutation remains unclear and should consequently be deeply studied. Another mutation, T206A, without any known role in virus infectivity or host tropism, distinguished our strains; such substitution was present only in the Korean-like H9N2 viruses but absent in the Moroccan strains isolated in 2016.

All the current Algerian strains shared the same glycosylation pattern with seven out of eight PGS, as compared to the G1-like prototype (Q/HK/G1/97). It has been reported that variations in PGS patterns of influenza virus may influence the pathogenicity, the affinity and the receptor binding specificity [36, 46] and possibly infection of a new host. Matrosovich et al., (1999) suggested that glycosylation patterns have a crucial role in the adaptation of avian influenza virus to poultry [47]. Besides, Reading et al., (2009) and Sun et al., (2013) have reported that a loss of PGS is associated with increased affinity of H5N1 and H1N1 subtypes to human-type receptors and with virulence in mice, respectively [48, 49]. In contrast, additional glycosylation sites on the HA protein were shown to decrease virulence [50].

The HB site of the NA protein contains six aa that interact directly with sialic acids; this includes three residues S at positions 367–372, 400 N, 403 W and 432 K [51]. Therefore, a change in these residues would alter sialic acid binding and host receptor specificity. The current Algerian isolates shared six substitutions in the NA HB site: S370L and S372A in the first loop, S400N, I402N and R403W in the second loop and Q432R in third loop, as compared to A/Quail/Hong Kong/G1/97 prototype; the latter showing the sequences ^366^IKKDSRSG^373^, ^399^DSDIRS^404^ and ^431^PQE^433^ in the three loops, respectively. The two substitutions (S372A and R403W) enhanced the cross of the species barrier and the adaptation to a mammalian host. These substitutions were also detected in H9N2 viruses isolated in Asia and the Middle East, and especially in H2N2 and H3N2 subtypes that caused human pandemics [41, 52]. These observations corroborate with the change in the binding specificity of HA protein of the Algerian strains to mammalian hosts. Finally, the substitution I402N, in the second loop of NA, offered one additional PGS to the Algerian strains (^402^NWS).

The Algerians H9N2 viruses showed additional PGS in the NA protein, as compared to G1-like virus A/Quail/Hong Kong/G1/97 (8–9 instead of 6 PGS). The PGS at position 402, characteristics of H9N2 viruses, was found in the Algerian isolates [53]. These observations showed that our strains may have developed an altered glycosylation pattern, which could consequently affect their pathogenicity [54] and increase their virulence [55] following alteration of the antigenicity or the sialidase activity [36] and the escape from the immune defenses [56]. Likewise, the increase in the glycosylation of NA protein of highly pathogenic strains promoted the cleavage of NA protein by cellular proteases and consequently the spread of the infection [57].

The NA inhibitors can link different aa residues at specific positions, in the enzyme active site, including at positions 118, 119, 151, 152, 274, 292, and 371. It has been reported that mutations E119G, R152K, H274Y, or R292K at the active site of NA, allowed for the development of drug resistance [7, 29, 58]. All Algerians strains are however predicted to be sensitive to neuraminidase inhibitors based on their NA sequences.

### Molecular characterization of internal proteins

Although mutations in the HA gene promote infection of novel hosts and mammalian adaptation, different studies have shown that internal proteins are also important and may affect host tropism and pathogenicity [59, 60]. Mutations within the replication complex genes (PB2, PB1, and/or PA) may induce enhanced virus replication [61]. The PA gene is considered as an important determinant in the virus adaptation to new hosts [62], and mutations on PA may increase virulence in animal models [63]. The RNP complex also form host-specific genetic lineage and signatures. In addition, the M1 protein (M1) interacts with the vRNP, and may determine the host range [64].

The PB2 protein in particular harbors different aa signatures associated with host range and efficient multiplication of AIV in mammals [60]; but, the most important is the aa at position 627. It is common that residues V, R, K and N at positions 588, 591, 627 and 701, respectively, are associated with increased virulence and adaptation to mammalian host. Algerian H9N2 strains harbored residues A, Q, E and D, which are associated with avian preferences [65–67]. In addition, the PB2 residues E158G [68], T271A [69], K339T [60], and T666I [70], which may lead to increased viral replication rates and pathogenicity in mammals, have not been detected in our strains. In fact, these latter shared 158E, 271 T and 339 K. However, the residue 702 K reported in the Tunisian H9N2 and Ck/HK/8733/01(H5N1) strains and considered as avian virus-like aa [13], was substituted by 702Q in the Algerian strains, with unknown function. Besides, the substitution K355R was also detected but was not associated with human associated H9 viruses [65]. While the substitution 627 K that confers high pathogenicity, virulence and increased replication in mice [63], was not detected in our Algerian viruses, three substitutions 318R, 590S and 661 T, associated with mammalian adaptation, were observed [71, 72]. Besides, the substitution 89 V that contributes to an increased polymerase activity in mouse cells was observed [73]. Additionally, the Algerian strains shared the substitutions 147 V and 504 V that are reported in the Moroccan and the Egyptian strains and associated with enhanced polymerase activity [74]**.** Molecular markers that were previously associated with avirulent strains such as 250 V [28], 253 D and 404 F, [75] were also observed in all Algerian studied strains (Table 5). Due to the many changes in the aa of PB2 of the studied strains, functional interactions between PB2 and other polymerase components might be affected [76]. Like adaptive mutations in polymerase activity of H1N1pdm09 and H7N9 viruses, increased significantly the activity of H9N2 in mammalian hosts [77]. It seems that combined mutations may increase the activity of H9N2 polymerase.

The PB1 substitutions N105S, K577E/M and 578Q, known to be associated with increased polymerase activity, H9N2 pathogenicity in mice as well as adaptation to mammalians [61, 64, 78], were not observed in the currently circulating Algerian strains, which however shared 105 N, 577 K and 578 K. Nevertheless, one molecular determinant (PB1 13P) of host specificity, adaptation to mice [64, 71] and enhanced polymerase activity [62], was detected. Similarly, one other substitution D662G, associated with increased polymerase activity and AIV H5N1 virulence in mice, has also been detected [79]. In addition, three substitutions R207K (polymerase activity in mammalian cells), H436Y (polymerase activity and virulence in mallards, ferrets and mice) and M677T (virulence related to mutation) [55], associated with high pathogenicity of H5N1, were also identified in Algerian sequences.

The influenza virus PA protein contain several aa substitutions that affect host range, polymerase activity and pathogenicity [60, 80]. Some aa, at crucial positions, may influence these functions and increase the risk for human infections [80, 81]. Algerian strains harbored three PA substitutions V100I, K312R and S409N, associated with adaptation to mammals [14, 28, 82] (Table 5). The unique aa substitutions such as V63I, T97I, K142N/E, S421I and R443K, which increase AIV polymerase activity, replication in mammalian cells and virus virulence in mice, were however not detected [61, 83–85]. Among the aa associated with increased virulence, PA protein of current Algerian strains shared three, 127 V, 550 L and 672 L, different molecular markers of virulence [14, 28, 59]. Likewise, the substitution T515A that was shown to reduce lethality of HPAIV H5N1 in avian species, polymerase activity in mammalian cells and virulence in duck [55], was absent. Instead, all Algerians strains exhibited the 515 T residue, associated with enhanced lethality. The studied strains retained the mutation 383D, which is highly conserved in avian influenza viruses and G-1 reference strain. Song et al., (2015) reported that this residue increases polymerase activity in mammalian and avian cell lines and may therefore allow species barrier crossing and human cases [86].

Influenza virus nucleoproteins have different aa residues that directly interact with the ARN polymerase, particularly with PB1 and PB2 sub-units [87]. NP has multiples functions in AIV biological cycle, replication/pathogenicity and mammalian host infection [88]. Different substitutions affecting NP functions have previously been reported. The aa sequence analyses of the Algerian H9N2 nucleoproteins showed conserved G-1 lineage residues 375D, 422R, 430 K, 442 T, 455D and 480D, associated with avian hosts [13, 59, 64, 82]. Zhu et al. (2015) reported that some substitutions in NP modulate the replication ability of G-1 virus in mammalian hosts. We also found that our isolates shared the aa Asp (D) at position 210, which is associated with increased polymerase activity in mammalian cells, and increased replication of H9N2 viruses especially at low temperatures [89]. Such mutations may therefore favor the replication of AIV H9N2 in the human upper respiratory tract. The NP substitution E372D was previously described for A/Chicken/Bangladesh/VP01/2006 (H9N2) [12] and considered as common in human associated H9 viruses [28, 59].

M1 and M2 proteins harbored different crucial sites related to host tropism, immune response [90] and resistance to antivirals M2 blockers [45]. The M1 molecular determinant of virulence 15I, conserved among H9N2 lineage [65] and associated with mammalian preference [28], were nevertheless observed. In addition, all strains possessed two residues, 30D and 215A, as reported in M1 of H9N2 isolated from human cases and known to increase virulence in mice [45]. Interestedly, the Algerians strain harbored a number of single aa substitutions at positions 30, 43 and 215, that were shown to contribute to the increase of virulence of HPAIV H5N1. Fang et al., (2009) reported that the substitution N30D and T215A, increase virulence and lethality in mice [91]. As compared to A/duck/Hokkaido/WZ83/2010 HPAIV H5N1 strain, aa sequence alignments showed that all Algerians strains shared the substitutions I43M [92], reported to increase virulence in mice, chickens and ducks. The M2 proteins of Algerian viruses retained the molecular marker 10 L of G-1 lineage and residues 11 T, 20S, 57Y, 78Q and 86 V shown to enhance avian infections [59, 82]. Analyses of other molecular markers of host range and virulence showed three M2 molecular markers 16D, 28 V and 55F, associated with mammalian transmission and human cases [82] and the two molecular virulence determinants, 64S and 69P [28]. The M2 protein is also considered as a target for antiviral drugs. Mutations in crucial positions can promote the development of antiviral resistance phenotype. Among aa substitutions associated with resistance to antiviral drugs, at positions 26, 27, 30, 31, 34 and 38, current Algerian strains only harbor one substitution (31 N), suggesting their resistance to amantadine and rimantadine [93, 94].

The NS1 protein of the Algerian strains are typical of H9N2 influenza virus with a PDZ (X-S/T-X-V)^227^GESV^230^ C-terminal motif, characteristic of avian species [13, 28]. Parvin et al., (2014) reported, however, that most AIV have ^227^ESEV^230^ (PDZ domain) at the end of their NS1 protein [12], increasing the virus virulence in mice [95]. Our strains exhibited the NS1 substitution E227G as compared to the G-1 like (227E) and the mammalian preference viruses (227 K/R) [59, 96]. The substitution E227G may indeed modulate the pathogenicity of the studied isolates, through structural change in the PDZ C-terminal motif, but their biological significance remains unknown. Algerian isolates shared the NS1 residue 42S [75, 97], which was shown to increase the virulence in mice and to decrease the antiviral immune response of host cells [97]. Two additional markers 106 M [98] and V149A [99], which increase viral replication in mammalian cell and virulence in mice and decrease interferon response in chicken, were detected.

## Conclusion

The present study reported, for the first time, the full-length genome sequences of ten AIV H9N2, recently isolated from a fatal influenza outbreak with respiratory manifestations that occurred in Northeast of Algeria, in April 2017. Phylogenetic and molecular analyses were used to study the full genome of these AIV isolates. Phylogenetic analysis showed that the surface and the internal genes have the same evolution patterns and are grouped with the Moroccan and the Middle Eastern strains, inside the G1- like H9N2. The Blast analysis demonstrated that all strains are also highly similar to the Algerian strains isolated during the same year, in the central region of Algeria (2017). These strains were similar to AIV H9N2 isolated in Morocco (2016). The implementation of adequate biosecurity measures to limit the introduction of the infection into the Algerian poultry farms is highly recommended.

Amino acids analysis showed that the Algerians H9N2 strains carried out different molecular markers associated with affinity to human-like receptors and increased virulence. Interestingly, different substitutions, common to H7N9 viruses and exclusive to the Algerian strains, were also revealed, indicating continuous evolution of AIV in the field. Therefore, further studies to monitor such strains evolution in the field and reduce the risk of virus transmission to humans are crucial. Additional studies using animal models are also required to investigate the pathogenicity of current Algerian H9N2 strains.

## Data Availability

Data supporting the conclusions of this article are presented in the manuscript.

## Abbreviations

aa: amino acid
AIV: Avian influenza viruses
aMPV: avian metapneumovirus
DMEM: Dulbecco’s Modified Eagle Medium
GTR: General Time reversible
HA: Hemagglutinin
HB: Heamadsorption
HPAIV: Highly pathogenic avian influenza virus
IBV: Infectious bronchitis virus
LPAIV: Low pathogenic avian influenza virus
M: Matrix
Min: Minute
NA: Neuraminidase
ML: Maximum likelihood
MS: *Mycoplasma gallisepticum*
NDV: Newcastle disease virus
NP: Nucleoprotein
NS: Nonstructural protein
nt: Nucleotide
Rpm: Round per minute
RT-PCR: Reverse transcription polymerase chain reaction
S: Second
PL: PDZ ligand
RBS: Receptor binding site
PA: Polymerase acidic
PB: Polymerase basic
PGS: Potential glycosylation sites
SPF: Specific pathogen free
